# Two-Step Operation for Aortoesophageal Fistula After Thoracic Endovascular Repair

**DOI:** 10.7759/cureus.67169

**Published:** 2024-08-19

**Authors:** Takeshi Ikuno, Yutaka Sakakibara, Yusuke Seki, Kazunobu Nishimura

**Affiliations:** 1 Department of Cardiac Surgery, Kyoto Medical Hospital, Kyoto, JPN; 2 Department of Cardiovascular Surgery, Takamatsu Red Cross Hospital, Takamatsu, JPN

**Keywords:** aortoesophageal fistula, descending aorta aneurysm, ruptured aneurysm, thoracic endovascular aortic repair, tevar

## Abstract

Aortoesophageal fistula (AEF) caused after thoracic endovascular aortic repair (TEVAR) is rare but a serious complication. We report a successful staged operation for AEF after TEVAR. A 70-year-old male underwent TEVAR for a ruptured aneurysm of the descending aorta and subsequently developed AEF three months later. First, the patient underwent the resection of the esophagus, which was the focus of the infection under the right thoracoscopic approach. Second, descending aorta replacement was performed using a left thoracotomy approach. The patient has been well for about two years since the second operation without recurring graft infection. Staged operation with a different approach to the infection zone is a useful method for AEF.

## Introduction

Aortoesophageal fistula (AEF) is one of the late complications of descending aorta replacement or thoracic endovascular aortic repair (TEVAR), with a frequency of 1.6% from 0.36% [1-3] but frequently has fatal progress with mortality of 25%-60% [1-11]. The early stage of AEF involves mild symptoms, such as vomiting or a positive fecal occult blood test, guiding the diagnosis of AEF and making it serious. AEF surely caused graft infection associated with a bad prognosis.

We described a successful staged operation for AEF after TEVAR. The esophagus was resected in the right thoracoscopic surgery. The descending aorta was then replaced with a left thoracotomy.

## Case presentation

**Figure 1:**
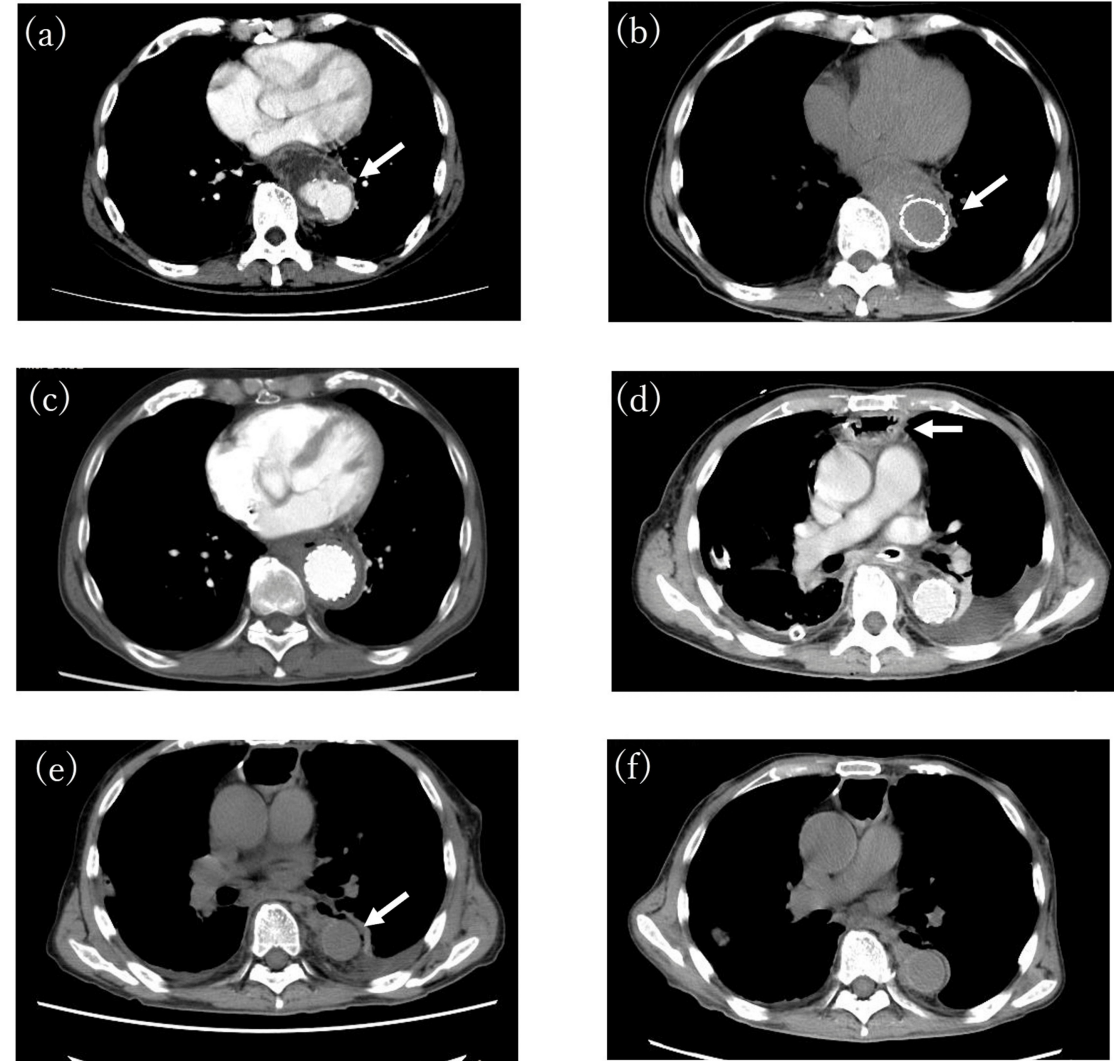
The time course of the axial-plane computed tomography (a) A 60-mm saccular pseudoaneurysm in the descending aorta (arrow); (b) After thoracic endovascular aortic repair (TEVAR) (arrow); (c) Two months after the TEVAR; (d) After esophageal reconstruction (arrow); (e) After descending aorta replacement (arrow)

**Figure 2:**
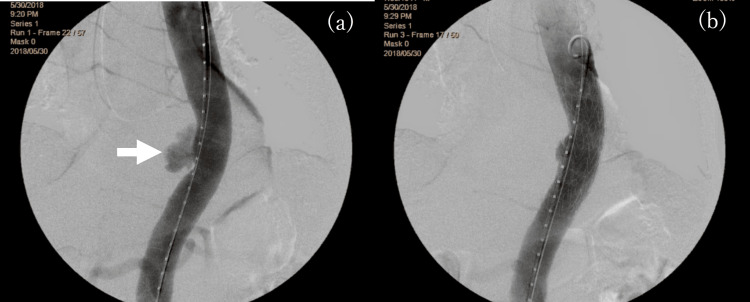
Aortography Aortography pre- (a) and postinsertion (b) of the stent graft for ruptured descending aorta aneurysm (arrow)

A 70-year-old man was admitted to the hospital with atypical mild back pain lasting for more than three weeks with a surgical treatment history for bladder cancer. The symptoms were mild, including normal body temperature. C-reactive protein concentration (5.5 mg/dL; normal range, <0.5 mg/dL) was only elevated; otherwise, white blood cell count (4.31 × 10^9^ /L; normal range, 4.419-12.68 × 10^9^ /L) and procalcitonin level (0.07 ng/ml; normal range, <0.5 ng/ml) were normal. However, computed tomography detected a saccular pseudoaneurysm of 60 mm in the descending aorta (Figure 1a). The patient was mildly frail (Clinical Frailty Scale 4), and he wanted to quickly recover after the operation. Therefore, our team decided to perform TEVAR. Urgent TEVAR was performed on the same day. The conformable TAG (cTAG 31-100 mm) thoracic stent graft (Gore Medical, Flagstaff, AZ, USA) was implanted in the descending aorta from the left common femoral artery (Figure 2). The surgery was successful and the patient was extubated in the operating room. The patient was discharged 12 days postoperatively.

**Figure 3:**
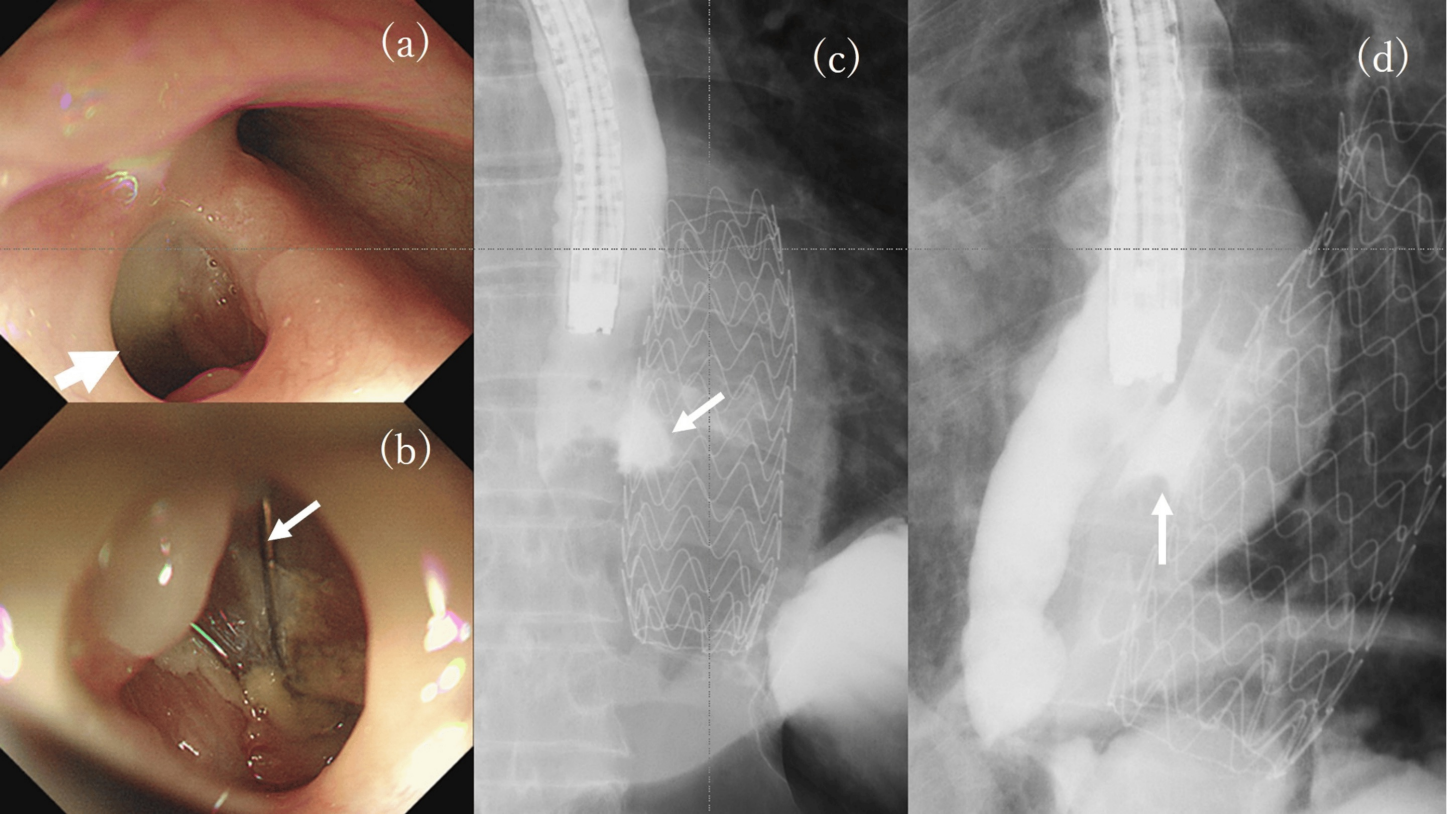
Diagnostic imaging of the aortoesophageal fistula (AEF) (a) (b) Upper gastrointestinal endoscopy finding; (c) (d) Esophagography with gastrografin; (a) A 10-mm left esophagus wall fistula at 30 cm from the incisors; (b) Stent graft appearance (arrow) through the fistula after washing; (c) (d) Contrasted AEF (arrow) by gastrografin around the stent graft

**Figure 4:**
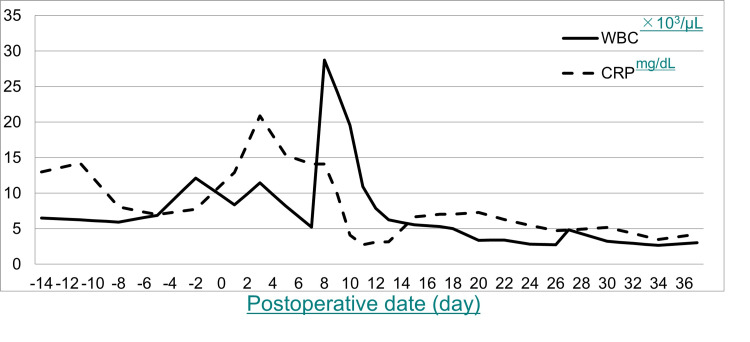
Time course of total leucocyte count (WBC) and C-reactive protein (CRP)

Trans-urethral resection of the prostate was performed for prostatic hypertrophy after two months. Simultaneously, the fever was prolonged and caused gastric distress. Upper gastrointestinal endoscopy revealed a 10-mm left esophagus wall fistula at 30 cm from the incisors. The stent graft was detected over the fistula when washing the AEF. Gastrografin was administered to confirm the expanse of the fistula and contrasted with an abscess cavity (Figure 3). Concurrently, the blood culture was negative. An antibiotic agent was changed to meropenem (MEPM) from ceftriaxone sodium (CTRX) as an empirical treatment for intestinal bacteria. The total leucocyte count was 250/μL and C-reactive protein (CRP) was 14.21 mg/dL, but this data revealed a total leucocyte count of 890/μL and CRP of 6.56 mg/dL after 10 days of MEPM administration (Figure 4). The fever has also improved.

We planned a two-stage operation. First, the esophagus, which was the source of the infection, is removed. Second, the infected stent graft is resected and a new woven graft is implanted. Concurrently, the first esophagectomy operation is executed under the tight thoracoscopic approach, whereas the second descending aortic aneurysm repair surgery is performed at the left anterolateral thoracotomy. Hence, the second operation could minimize adhesion and reduce invasion.

**Figure 5:**
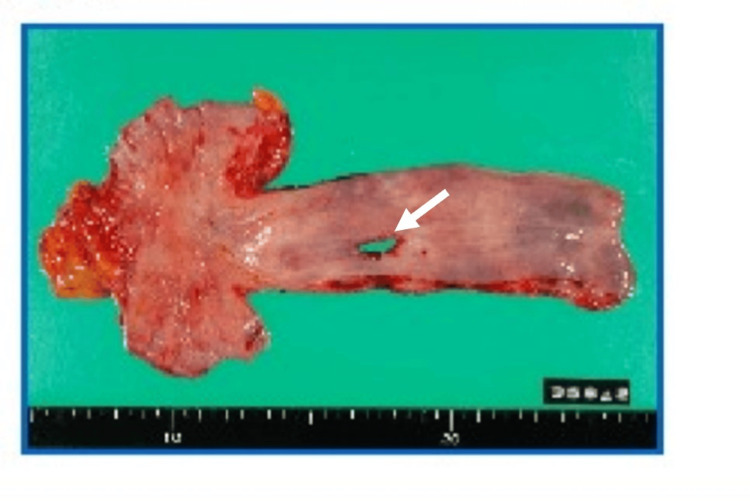
Resected esophagus with the aortoesophageal fistula

Thoracoscopic esophageal resection and reconstruction were performed. The descending aorta exhibited severe adhesion because of the inserted stent and the inflammation. The esophagus was isolated from this adhesion, pus was discharged, and the fistula formation was identified (Figure 5). The thoracoscopic gastric tube reconstruction using the retro-sternal route was performed after laparoscopic proximal gastrectomy. The omentum was fixed in the left upper part of the abdominal cavity to implant the left thoracic cavity when preparing for the next surgery. Additionally, Enterobacter cloacae and a Gram-positive bacillus were detected in the culture of abscesses collected around the stent graft. The abscess was aspirated using a drainage tube, but the pus discharge was continuously drained.

Descending aorta replacement was performed a week after the esophageal reconstruction. A left thoracotomy was performed using the modified straight incision with rib-cross (SIRC) approach [12-13]. The left femoral artery and vein were cannulated to create a cardiopulmonary bypass. The patient underwent a descending aorta replacement to a rifampicin-soaked vascular prosthesis with proximal descending aorta clamping during moderate hypothermia. The pedicled omentum was wrapped in the new woven graft after fenestrating the diaphragm. The infected stent graft was removed, and the infected native descending aorta was resected. Enterococcus and Corynebacterium caused this abscess. The bacteria were detected from the abscess around the stent graft, the left hydrothorax, and the native descending aorta. Corynebacterium infection was confirmed and vancomycin was administrated more because of MEPM resistance. After the second operation, four weeks of additional antibiotic treatment was given under the 2017 American Heart Association/American College of Cardiology (AHA/ACC) guidelines on infective endocarditis, which is a similar field for cardiovascular infection. The patient recovered quickly, and after two days in the intensive care unit, he was transferred to the general ward. The patient was discharged 60 days after the last surgical procedure. No recurrence of any infection was observed at the two-year follow-up.

## Discussion

AEF is one of the complications of midterm and long-term results after surgical descending aorta replacement or TEVAR. The incidence of AEF to 1.6% from 0.36% [1-3]. However, the number of AEF case reports is high in the TEVAR era [1-5,14,15]. Czerny et al. suggested that AEF was associated with the need for emergency TEVAR and mediastinal hematoma formation [14]. Takei et al. reported that once an endoleak occurs, aortic aneurysms gradually increase in size, and continued pressurization of the aneurysm sac may predispose patients to AEF formation [15].

AEF after any aortic intervention is a devastating and life-threatening condition, with a fatality rate of 25%-60% [1-11]. Cruel mortality involved two reasons. The first reason is a very high surgical risk in the operations patients with post-interventional AEF need [1,14]. Reoperation in the same surgical approach as the initial surgery augments the difficulty due to thick adhesion and a larger risk of bleeding and infection. The second reason is an essential risk of recurrent prosthetic graft infection, even though the patients survive the radical operations [15-16]. Recently, various solutions have been considered and reported for this problem [6,17,18]. The mortality of this radical operation was unclear because of its rarity. Few recent reports have indicated a success rate of surgical treatment, which was far from satisfactory [4,12], thereby reducing operation aggression and controlling infection. Deciding to perform surgery in one step or stage, and controlling the infection, is important. We emphasize several key points. The operation should be highly invasive and take a long time because this one-step operation treats an infective aneurysm after esophagectomy. In that situation, an extra-anatomic bypass might be an alternative. One-step surgery with an extra-anatomic bypass does not eliminate the recurrence of a graft infection perfectly. However, an extra-anatomic bypass avoids the implantation of a prosthesis in the same infection site. In addition, the extra-anatomic bypass further increases surgical invasion, and it could be at risk of insufficient blood perfusion and long-term graft failure. Hence, we currently planned a two-step surgery comprising esophageal reconstruction and thoracic aortic replacement using an entirely different approach. First, we performed esophagectomy and upper gastrointestinal tract reconstruction, which involves an omental flap for the infectious lesion, in right thoracoscopic surgery. The first surgery was uneventfully completed with an adequate operation time and a relatively low risk. The descending aorta was then replaced after excising the infected aneurysm, and a TEVAR graft was performed via the left thoracotomy. We localized infection only to the infected TEVAR graft by removing the esophageal fistula that was the source of infection as well as draining the abscess at the time of the initial surgery. The second surgery exhibited no thick adhesions in the left thorax, whereas the localized infectious lesion was limited to the TEVAR graft.

To reduce the surgical invasion and minimize the risk of recurrent infection, we performed a two-step bilateral thoracotomy approach, namely, the first right thoracoscopic esophagectomy and the second descending aortic replacement via the left thoracotomy. The strategies in this complicated AEF case were extremely useful. Even now, five years later, the patient is followed up at our outpatient clinic with no recurrent graft infection at all.

## Conclusions

AEF is a rare but severe complication of TEVAR. AEF was detected following TEVAR for a descending aorta ruptured aneurysm. We performed a successful two-step operation for infection control and invasion regression. The thoracic cavity approach avoids infectious mediastinitis. First, the right thoracoscopic esophagectomy prevented the spread of bacterial infection. Then, the second localized infected aneurysm was safely resected.
